# Acute Stroke in Diabetes Mellitus: A Prospective Observational Study Evaluating the Course and Short-Term Outcome in Basrah, Southern Iraq

**DOI:** 10.7759/cureus.6017

**Published:** 2019-10-28

**Authors:** Mahmood Thamer Altemimi, Ali Raheem Hashim

**Affiliations:** 1 Endocrinology, Faiha Specialized Diabetes, Endocrine and Metabolism Center, Basrah, IRQ; 2 Medicine, Basrah Teaching Hospital, College of Medicine, University of Basrah, Basrah, IRQ

**Keywords:** stroke, diabetes mellitus, aspirin failure, national institutes of health stroke scale (nihss)

## Abstract

Introduction

Stroke and diabetes mellitus (DM) are distinct conditions with many aspects in common. Both conditions are prevalent and associated with various vascular risk factors such as dyslipidemia and hypertension. This study was conducted to evaluate the association between stroke and DM regarding the course (i.e., type, recurrence, aspirin failure, and degree of disability) of stroke and short-term outcomes in patients with diabetes who suffer an acute stroke.

Patients and methods

We conducted a prospective observational study of 210 acute stroke patients admitted to the Neurology Unit of Al-Basrah Teaching Hospital in Basrah City in Southern Iraq from May 2014 to September 2015. Our study included 124 men (59%) and 86 (41%) women, and our study population had a mean age of 64 ± 11 years. The study participants were separated into two groups of 105 patients according to their diabetes status, and we evaluated each patient’s type of stroke, recurrence, aspirin failure, and degree of neurological disability according to the National Institutes of Health stroke scale. We assessed short-term outcomes (i.e., four weeks' mortality) following acute stroke.

Results

Ischemic stroke was the primary stroke experienced by patients with diabetes (94.3%), which is highly significant and associated with an increased risk of previous recurrent ischemic stroke (55.2%; P=0.003, <0.001, respectively). We noted a higher rate of loss of antiplatelet activity from aspirin in patients with DM rather than those without DM (p<0.001), and we noted more severe disability in outcomes in patients with DM. In the group of stroke patients with diabetes, 46 patients (59%) died, while 32 patients (41%) without diabetes died (p=0.046).

Conclusion

DM is associated with a heavier burden for acute stroke along with poorer outcomes than patients without DM. Our findings suggest acute stroke in patients with diabetes may be considered a distinct entity separate from acute stroke in patients without diabetes. The treatment of acute stroke in patients with diabetes warrants further investigation.

## Introduction

Stroke and diabetes mellitus (DM) are two prevalent conditions that share several risk factors, such as hypertension and dyslipidemia [1]. The prevalence of type-2 diabetes mellitus (T2DM) is increasing in both developing and developed countries [2]. In Basrah, a city in Southern Iraq, the prevalence of T2DM has increased significantly with age from 3.4% among patients aged 19 to 30 years to approximately 43% among adults aged 46 to 60 years [3].

T2DM nearly doubles the risk of stroke, and substantially increases the risk of first ischemic stroke; DM may be responsible for more than 8% of first ischemic strokes [4-6]. The effect of prediabetes on the prognosis of acute stroke patients has not been adequately studied, but T2DM itself is associated with increased risk for recurrent ischemic stroke [7]. T2DM raised the rate of recurrent stroke, deterioration of neurological deficit, enlargement of ischemic brain lesion, and increased frequency of complications after stroke (e.g., multiple organ dysfunction and urinary tract infections) [8].

This study was conducted to examine the association between stroke and T2DM concerning the course of stroke (including the type of stroke, history of recurrence, aspirin failure, and degree of neurological disability) and short-term outcomes (i.e., four-week mortality). We also explored the possibility of stroke in the context of T2DM as deserving of a separate medical status than stroke in the absence of T2DM.

## Materials and methods

The study population consisted of patients admitted in the Neurology Unit of Al-Basrah Teaching Hospital in Basrah, Southern Iraq, from May 2014 to September 2015. All patients were diagnosed with acute stroke according to an updated definition from the American Heart Association and American Stroke Association [9]. Patients were excluded from the study if they had blood dyscrasia or other hematological disorders such as hemoglobinopathies, were on warfarin therapy, had congenital or valvular heart disease, were pregnant, had connective tissue diseases, malignancy, transient ischemic attack, neurological deficits due to hypoglycemia, atrial fibrillation, hemiplegic or basilar migraine, or chronic kidney disease (with or without renal replacement therapy).

Two hundred ten patients with acute stroke were enrolled in this prospective observational study, of whom 124 (59%) were men and 86 (41%) were women. The mean age of the study population was 64 ± 11 years. Patients were classified into two groups consisting of those with T2DM and those without T2DM. Patients were placed in the T2DM group if they used hypoglycemic medications (e.g., insulin or sulfonylureas), reported a physician diagnosis of diabetes, or had laboratory criteria meeting the threshold of T2DM according to the American Diabetes Association (ADA) [10].

We collected patient’s baseline information at the time of admission which consisted of demographic data (e.g., gender, age, residence, occupation, and level of education) and clinical risk factors including hypertension (history of hypertension or hypertensive drug use), dyslipidemia (history of dyslipidemia or having abnormal laboratory lipid profile), atrial fibrillation (the presence of the arrhythmia during hospitalization, or history of atrial fibrillation confirmed by at least one electrocardiogram [ECG]), and coronary artery disease (evidence of ischemic heart disease [IHD] documented by at least one ECG, or a medical report of IHD) [11]. We also collected data on history of aspirin medication use (defined as the use of low-dose aspirin for primary or secondary prevention of vascular events before the attack of acute stroke and patient smoking status (defined as having smoked ≥100 cigarettes over their lifetime and responded "every day" or "some days" to the question, "Do you now smoke cigarettes every day, some days, or not at all?") [12,13]. We also collected data on the history of alcohol consumption, known cases of thyroid disease, connective tissue disease, and female patient use of oral contraceptives. Finally, we collected data on history of previous stroke as recurrence, documented when a patient’s medical record confirmed the history of stroke by aggravated primary neurologic deficit, new signs, or rehospitalization due to any type of stroke [14].

Examination

All patients were examined for pulse rate, upper limb blood pressure measurement (in mm Hg), precordium examination, waist circumference measurement in centimeters (central obesity was documented when patient waist circumference was more than 88 cm for women and more than 102 cm for men according to the International Diabetes Federation) [15].

Neurologic disability was evaluated by the National Institutes of Health Stroke Scale (NIHSS) within 24 hours after admission. According to this scale, acute stroke patients were assessed as having a minor-to-moderate disability when their NIHSS score was less than or equal to 15, and those with an NIHSS more than 15 were considered severely disabled [16].

Laboratory tests

Laboratory investigations consisted of a renal function test, complete blood count, and brain imaging study in the form of CT scan and/or MRI. Fasting plasma glucose (FPG), glycated hemoglobin (HbA1c), and fasting serum lipid profile (low-density lipoprotein cholesterol [LDL-C] and high-density lipoprotein cholesterol [HDL-C]) were measured in the early morning after at least eight to 10 hours of fasting using 5 mL venous blood. Measurements were performed using a fully-automated clinical chemistry analyzer, the COBAS INTEGRA® 400 plus (Roche Diagnostics, Basel, Switzerland). An abnormal lipid profile was defined as LDL-C more than 100 mg/dL and HDL-C less than 40 mg/dL for men or less than 50 mg/dL for women [11].

According to the ADA, FPG ≥126 mg/dL is considered abnormal. We measured HbA1c for patients with T2DM and those without T2DM but had an abnormal FPG. HbA1c ≥6.5% was diagnostic of T2DM in non-diabetic patients, and HbA1c ≥7% was considered uncontrolled T2DM for acute stroke patients. All enrolled patients were monitored via follow-up after four weeks, and in the event of death, cause of death was documented.

Statistical analysis

We used IBM SPSS Statistics for Windows, Version 22.0 (IBM Corp., Armonk, NY) for data analysis. Descriptive analysis was done using mean ± standard deviation, frequency, and percentage of each value with the chi-squared test, and p<0.05 was considered significant.

## Results

Of the 210 patients enrolled in the study, 188 patients (89.5%) lived in urban areas, while 22 (10.5%) lived in rural districts. According to their level of education, there were 193 (91.9%) patients who did not complete their secondary school, and 17 (8.1%) patients completed their secondary school degree or higher. Our study population’s occupations were homemaker (n=85; 40.5%), self-employed (n=56; 26.7%), employee (n=21; 10%), and retired (n=48; 22.9%) (Table 1). We found no significant differences between the diabetes and non-diabetes group regarding residence, level of education, and occupations.

**Table 1:** Comparative characteristics of acute stroke patients with and without T2DM. Abbreviations: T2DM, type-2 diabetes mellitus; FPG, fasting plasma glucose; HT, hypertension; LDL-C, low-density lipoprotein cholesterol; HDL-C, high-density lipoprotein cholesterol.

Variables		Not-DM	T2DM	Total	p-value
Gender	Men	69 (65.7%)	55 (52.4%)	124 (59%)	0.049
	women	36 (34.3%)	50 (47.6%)	86 (41%)	
Age (years)	Mean ± SD	63.7±10.24	65.5±12.97	64.6 ±11.69	0.43
	≤50	17 (16.2%)	13 (12.4%)	30 (14.3%)	
	>50	(83.8%)	92(87.6%)	180 (85.7%)	
Address	Urban	95 (90.5%)	93 (88.6%)	188 (89.5%	0.652
	Rural	10 (9.5%)	12 (11.4%)	22 (10.5%	
Education	Less than secondary	93 (88.6%)	100 (95.2%)	193 (91.9%)	0.077
	Secondary and above	12 (11.4%)	5 (4.8%)	17 (8.1%)	
Occupation	Homemaker	36 (34.3%)	49 (46.7%)	85 (40.5%)	0.127
	Self-employed	30 (28.6%)	26 (24.8%)	56 (26.7%)	
	Employee	9 (8.6%)	12 (11.4%)	21 (10%)	
	Retired	30 (28.6%)	18 (17.1%)	48 (22.9%)	
History of HT	Not HT	32 (30.5%)	18 (17.1%)	50 (23.8%)	0.023
	HT	73 (69.5%)	87 (82.9%)	160 (76.2%)	
Smoking	No smoking	59 (56.2%)	68 (64.8%)	127 (60.5%)	0.204
	Smoker	46 (43.8%)	37 (35.2%)	83 (39.5%)	
Other Risk Factors	No	52 (49.5%)	52 (49.5%)	104 (49.5%)	1.000
	Yes	53 (50.5%)	53 (50.5%)	106 (50.5%)	
LDL-C (mg/dL)	Mean ± SD	128.3 ± 44.82	136.6 ± 48.89	132.4 ± 46.97	0.758
	Normal	30 (28.6%)	28 (26.7%)	58 (27.6%)	
	High	75 (71.4%)	77 (73.3%)	152 (72.4%)	
HDL-C (mg/dL)	Mean ± SD	43.43 ± 15	39.02 ± 11.74	41.22 ± 13.6	0.030
	Normal	45 (42.9%)	25 (23.8%)	70 (33.3%)	
	Decrease	60 (57.1%)	80 (76.2%)	140 (66.7%)	
FPG (mg/dL)	Mean ± SD	116.8 ± 29.4	227.6 ± 92.26	172 ± 88.02	<0.001
	Normal	77 (73.3%)	16 (15.2%)	93 (44.3%)	
	Abnormal	28 (26.7%)	89 (84.8%)	117 (55.7%)	
Obesity Rating Based on Waist Circumference	Mean ± SD	95.35 ± 11.04	97.11 ± 11.23	96.2 ± 11.14	0.167
	Normal	60 (57.1%)	50 (47.6%)	110 (52.4%)	
	Central Obesity	45 (42.9%)	55 (52.4%)	100 (47.6%)	
Total		105	105	210	

A total of 160 (76.2%) patients had chronic hypertension. Eighty-three (39.5%) patients had a history of smoking, and there were 106 (50.5%) patients with at least a single additional risk factor such as central obesity, dyslipidemia, coronary artery disease, alcohol consumption or female using oral contraceptives (Table 1).

For comparative characteristics of acute stroke patients, we found gender, history of hypertension, FPG and HDL-C levels were associated with significant differences between patients with T2DM and those without (p=0.049, 0.023, 0.030, and <0.001, respectively) while other parameters yielded no significant differences (Table 1). In comparison with the non-diabetic group, T2DM increased the chances of an acute stroke among older patients; however, this was not statistically significant (p=0.43; Table 1).

This study enrolled 105 patients with diabetes; 55 (52.4%) were men, and 50 (47.6%) were women. The mean age of the diabetes group patients was 63 ± 10 years, and their mean FPG was 227.6 ± 92.26 mg/dL. Thirteen patients with T2DM (12.4%) were aged 50 years or younger, while 92 (87.6%) patients were older than 50 years. Ninety-one (86.7%) patients had T2DM before the incidence of an acute stroke, while 14 (13.3%) patients were newly diagnosed with T2DM according to their laboratory reading and ADA thresholds. An elevated FPG was more obvious among the patients with T2DM compared to those without diabetes (p<0.001), and 26.7% of patients without diabetes had elevated FPG due to stress hyperglycemia (i.e., an acute illness may transiently disturb measures of plasma glucose) [17]. According to waist circumference measurements, 55 (52.4%) patients had central obesity as compared with 50 (47.6%) patients with waist circumference within reference limits. Seventy-seven patients (73.3%) had abnormal LDL-C levels, while 28 (26.7%) had LDL-C levels within reference limits. The mean LDL-C was 136.6 ± 48.89 mg/dL. Eighty (76.2%) patients had low levels of HDL-C, while 25 (23.8%) patients had HDL-C level within reference limits. The mean HDL-C was 39.02 ±11.74 mg/dL (Table 1).

In the diabetes group, the most common stroke was ischemic (94.3%). We noted a significant association in the incidence of a first ischemic type of stroke in patients with T2DM as compared with those without diabetes (p=0.003; Table 2). There was a highly significant association between history of previous stroke recurrence and T2DM in contrast to those without DM (p<0.001). A high rate of loss of antiplatelet activity of aspirin was documented in patients with diabetes compared with the group without diabetes (p<0.001; Table 2). The degree of neurological disability of acute stroke as assessed by NIHSS was more severe among diabetic patients compared with non-diabetic patients (p=0.042; Table 2).

**Table 2:** Distribution of clinical course of acute stroke between patients with and without T2DM Abbreviations: T2DM, type-2 diabetes mellitus; NIHSS, National Institutes of Health Stroke Scale.

Clinical Parameters		Not-DM	T2DM	Total	p-value
Type of Stroke	Ischemic Stroke	85 (81%)	99 (94.3%)	184 (87.6%)	0.003
	Hemorrhagic Stroke	20 (19%)	6 (5.7%)	26 (12.4%)	
History of recurrent stroke	No	73 (69.5%)	47 (44.8%)	120 (57.1%)	< 0.001
	Yes	32 (30.5%)	58 (55.2%)	90 (42.9%)	
History of aspirin use	No	94 (89.5%)	72 (68.6%)	166 (79%)	< 0.001
	Yes	11 (10.5%)	33 (31.4%)	44 (21%)	
NIHSS	Minor/moderate	76 (72.4%)	62 (59%)	138 (65.7%)	0.042
	Severe	29 (27.6%)	43 (41%)	72 (34.3%)	
Total		105	105	210	

In patients with T2DM, the mean duration of T2DM was 7 ± 5 years, and 48 (45.7%) patients had T2DM for five years or less, while 57 (54.3%) patients had T2DM for more than five years. The mean HbA1c for T2DM was 9.08% ± 1.89% (Table 3). Sixteen (15.2%) patients had a good degree of glycemic control by HbA1c ≤ 7%, while 89 (84.8%) patients were uncontrolled (Table 3). A greater level of neurological disability was documented among the uncontrolled T2DM (i.e., those with HbA1c >7%) rather than those with good glycemic control (HbA1c ≤ 7%; p=0.043; Table 3).

**Table 3:** Association between degree of neurological deficit according to the NIHSS and HbA1c with duration of T2DM in acute stroke Abbreviations: T2DM, type-2 diabetes mellitus; HbA1c, glycated hemoglobin; NIHSS, National Institutes of Health Stroke Scale.

T2DM patients		NIHSS		Total	P value
		Minor/moderate	severe		
HbA1C	Mean ± SD	-	-	9.08 ± 1.89	0.043
	Hb-A1c ≤ 7%	13 (81.3 %)	3 (18.8%)	16 (15.2%)	
	Hb-A1c > 7%	49 (55.1 %)	40(44.9%)	89 (84.8%)	
Duration of T2DM (years)	Mean ± SD	-	-	7.23 ±5.29	0.509
	≤ 5 years	30 (48.4%)	18 (41.9%)	48 (45.7%)	
	>5 years	32 (51.6%)	25 (58.1%)	57 (54.3%)	
Total		62	43	105	

When we assessed the effect of T2DM on the short-term outcome of acute stroke (i.e., four-week mortality documentation), a high rate of death registered among stroke patients with T2DM compared to stroke patients without diabetes (p=0.046; Table 4).

**Table 4:** Association of demographic and clinical parameters on short-term outcome (four-week mortality) in acute stroke Abbreviations: T2DM, type-2 diabetes mellitus; HT, hypertension; NIHSS, National Institutes of Health Stroke Scale.

Parameters		Outcome		Total	p-value
		Died	Alive		
Gender	Men	30 (38.5%)	94 (71.2%)	124 (59%)	<0.001
	Women	48 (61.5%)	38 (28.8%)	86 (41%)	
Age (years)	≤50	3 (3.8%)	27 (20.5%)	30 (14.3%)	<0.001
	>50	75 (96.2%)	105 (79.5%)	180 (85.7%)	
Smoking	No smoking	57 (73.1%)	70 (53.0%)	127 (60.5%)	0.004
	Smoker	21 (26.9%)	62 (47.0%)	83 (39.5%)	
History of HT	Not HT	18 (23.1%)	32 (24.2%)	50 (23.8%)	0.848
	HT	60 (76.9%)	100 (75.8%)	160 (76.2%)	
T2DM	Not DM	32 (41.02%)	73 (55.3%)	105 (50%)	0.046
	T2DM	46 (58.98%)	59 (44.69%)	105 (50%)	
Type of Stroke	Ischemic	73 (93.6%)	111 (84.1%)	184 (87.6%)	0.043
	Hemorrhagic	5 (6.4%)	21 (15.9%)	26 (12.4%)	
History of Recurrent Stroke	No	28 (35.9%)	92 (69.7%)	120 (57.1%)	<0.001
	Yes	50 (64.1%)	40 (30.3%)	90 (42.9%)	
History of Aspirin Use	No	49 (62.8%)	117 (88.6%)	166 (79.0%)	<0.001
	Yes	29 (37.2%)	15 (11.4%)	44 (21.0%)	
NIHSS	≤ 15	11 (14.1%)	127 (96.2%)	138 (65.7%)	<0.001
	>15	67 (85.9%)	5 (3.8%)	72 (34.3%)	
Total		78	132	210	

The four-week mortality rate increased with increasing age in both men and women, but women experienced a higher mortality rate than men (61.5% for women versus 38.5% for men), especially among the older participants. Other factors such as smoking history, type of stroke, history of recurrent stroke, history of aspirin use, and high NIHSS score for neurological disability also significantly affect the short-term outcome for acute stroke patients (Table 4). However, after doing a multiple logistic regression analysis to all participants (Table 5), T2DM has an independent and significant effect on short-term outcomes along with the degree of disability and aspirin use.

**Table 5:** Binary logistic regression for variables in regard short-term outcome (four-week mortality) of all acute stroke Abbreviations: T2DM, type-2 diabetes mellitus; HT, hypertension; BP, blood pressure; FPG, fasting plasma glucose; NIHSS, National Institutes of Health Stroke Scale; LDL-C, low-density lipoprotein cholesterol; HDL-C, high-density lipoprotein cholesterol; B, unstandardized regression weight; SE, standard error; Wald, Wald test, chi-squared statistics to test the significance of individual coefficients in the model; df, degree of freedom; Sig, significance; Exp(B), exponentiation of the coefficients.

Variables	B	SE	Wald	df	Sig	Exp(B)
Age	-.067	.059	1.299	1	.254	.935
Gender	-2.146	1.403	2.340	1	.126	.117
Education	1.349	2.998	.203	1	.653	3.855
T2DM	7.787	3.699	4.431	1	.035	2408.033
Duration of T2DM	-.011	.171	.004	1	.947	.989
HT	.977	1.660	.347	1	.556	2.658
Smoking History	-.260	1.290	.041	1	.840	.771
Other Risk Factors	.372	1.158	.103	1	.748	1.451
Types of Stroke	-2.748	1.993	1.901	1	.168	.064
LDL-C	.008	.009	.751	1	.386	1.008
HDL-C	.038	.045	.692	1	.406	1.038
Recurrence	.408	1.447	.080	1	.778	1.504
NIHSS	-1.153	.282	16.664	1	.0001	.316
BP	-.689	1.177	.342	1	.558	.502
FPG	.012	.008	2.471	1	.116	1.012
Aspirin Use	-3.588	1.596	5.052	1	.025	.028
Constant	22.902	9.372	5.972	1	.015	8838243053.970

## Discussion

The age demographics of our patient population aligned with that reported in many WHO Eastern Mediterranean Countries [18]. The male to female ratio in our study was 1.44:1, which is similar to other male preponderant studies [18,19]. Being female with diabetes carried a more significant stroke burden than being male with diabetes, but this could be explained by the fact that women tend to live longer than men [19-21]. The low HDL-C levels were linked to risk of ischemic stroke and elevated lipoprotein (a), which is also related to the incidence of stroke [22].

In this study, ischemic strokes were the most common (87.6%), which aligns with previous reports of the burden of ischemic stroke worldwide (75% to 85%) [19]. The risk of recurrent ischemic stroke in this study was similar to the Cardiovascular Health Study findings that reported a 60% increased risk for recurrence in patients with first ischemic stroke [23]. This study reveals that the clinical efficacy of aspirin was reduced in the T2DM population, further supporting the idea that a state of hyperglycemia interferes with aspirin acetylation due to the excessive glycation of platelets and coagulation factors [24].

The neurological disability was significantly more frequent in stroke patients with T2DM than in those without T2DM, indicating that T2DM is a strong determinant for dependency in acute stroke patients. These results were inconsistent with many previous studies [25,26]. In T2DM, there was a significant association between the degree of neurological disability and plasma glucose control status. The worse dependency found among patients with uncontrolled diabetes in comparison with those with healthy glycemic control was similar to other studies, which suggests a deleterious effect for acute stroke patients with high HbA1c [27].

The four-week mortality rate of stroke was higher than the average worldwide mortality rate of 22.9%, but it was similar to the mortality rate reported in some Western and Asian countries such as Bulgaria, Georgia, and Iran [19]. A possible explanation for the discrepancy in mortality rates may be due to the lack of specialized stroke units in Basrah. The low socioeconomic status of diabetes patients may also disturb glycemic control in this locality as compared with others. The four-week mortality finding was not surprising due to the double stroke risk for both sexes for every successive ten years after age 55 [24]. Smoking history was inversely associated with short-term outcomes as the majority of the stroke patients who died were non-smoking women.

T2DM, ischemic stroke, history of recurrent stroke, history of aspirin use, and degree of disability as assessed by NIHSS significantly affected the four-week mortality of acute stroke. T2DM worsened the course of a stroke by influencing the type, recurrence, aspirin failure, and dependency of acute stroke; therefore, acute stroke could be considered a distinct entity in patients with T2DM.

The study was carried out in the absence of several important pieces of equipment that limited our ability to assess certain factors in acute stroke patients such as in-bed patient weight and telemetry recording to exclude undocumented arrhythmias. Future studies that can account for these factors will further elucidate the relationship between T2DM and acute stroke outcomes.

## Conclusions

T2DM is associated with a worse burden on the course of acute stroke. Proper glycemic control with HbA1c <7% yields a better neurological course in acute stroke patients than poor glycemic control. T2DM remains an independent predictor for poor outcomes in stroke patients. Acute stroke in patients with T2DM may be considered a distinct entity separate from an acute stroke in patients without diabetes.
